# Histopathologic and Molecular Features of Cutaneous Melanoma in a Moroccan Population

**DOI:** 10.7759/cureus.42691

**Published:** 2023-07-30

**Authors:** Layla Tahiri Elousrouti, Nawal Hammas, Imane Fadlallah, Sanae Elberdai, Iamiae Amaadour, Sara Elloudi, Fatima Zahra Elmernissi, Mohamed Elidrissi, Wissal Hassani, Badr Alami, Laila Chbani

**Affiliations:** 1 Department of Pathology, Biomedical and Translational Research Laboratory, Faculty of Medicine and Pharmacy, Sidi Mohamed Ben Abdellah University, Fez, MAR; 2 Department of Pathology, Hassan II University Hospital, Fez, MAR; 3 Department of Medical Oncology, Hassan II University Hospital, Fez, MAR; 4 Department of Dermatology, Hassan II University Hospital, Fez, MAR; 5 Department of Orthopedic Surgery, Hassan II University Hospital, Fez, MAR; 6 Department of Radiation Oncology, Hassan II University Hospital, Fez, MAR; 7 Department of Radiology, Hassan II University Hospital, Fez, MAR

**Keywords:** skin melanoma, survival rate, c-kit, braf v600e mutation, acral lentiginous

## Abstract

Background

Cutaneous cancer is the most common malignancy type, among which melanomas are considered the most aggressive and lethal. In Morocco, skin melanoma is the 25th most common cancer. To our knowledge, this is the first and largest Moroccan study specifically describing cutaneous melanoma.

Materials and methods

We obtained data for 100 patients diagnosed with cutaneous melanoma in the Department of Pathology of Hassan II University Hospital, Morocco. Clinical, histopathological, molecular, and follow-up data were recorded from pathology request forms and the patient’s medical records.

Results

The mean age of our patients was 65 years old. Histologically, the most prevalent were the nodular (48%) and acro-lentiginous (38%) melanoma subtypes. A total of 66% of the patients had a Breslow thickness of >4 mm. The presence of ulceration was noted in 46% of cases. The average mitoses was 9/1 mm². A total of 44% of patients had metastatic melanoma at the time of diagnosis. The BRAF V600E mutation was found in six cases, and the C-KIT mutation in five cases. The five-year overall survival and metastasis-free survival were 85% and 15%, respectively. There was a significant correlation between Breslow thickness and Clark’s level (*p*<0.001), histologic subtype (*p*=0.012), and presence of metastasis (*p*=0.002). There was a significant difference between the head and neck melanomas and those of the feet, particularly in the histological subtype and the presence of ulceration. BRAF V600E mutation was found in six cases of metastatic melanomas of the head and neck, of which three cases were positive for this mutation, as compared with the 23 cases of acral melanomas, which tested negative for the same mutation.

Conclusion

The results of our study showed that cutaneous melanomas were characterized by advanced age at diagnosis and late-stage diagnosis with a high Breslow index. The lower limbs were the most affected sites, especially in the plantar region. The acral lentiginous subtype was the most common. The presence of BRAF V600E mutation was associated with a better prognosis.

## Introduction

Cutaneous melanoma, one of the most aggressive forms of skin malignancy, is a malignant tumor formed from melanocytes [1]. According to Globocan 2020 data, skin melanoma ranks 17th worldwide for the number of cancer cases, accounting for 1.8% of new cases diagnosed in 2020 and 0.6% of deaths [2]. In Morocco, skin melanoma was shown to be the 25th most common cancer, with 248 new cases in 2020, accounting for 0.42% of new cases and 114 (0.32%) deaths [3]. Exposure to ultraviolet (UV) light has been shown to correlate with melanoma risk. However, the number of melanocytic nevi, family history, and genetic susceptibility are the most influential risk factors associated with this malignancy [4]. In the fourth edition of the 2018 WHO classification of skin tumors, melanomas are classified according to the degree of sun exposure. Therefore, melanomas resulting from different levels of sun exposure are differentiated. This includes melanomas with a low degree of cumulative sun damage, such as superficial spreading melanoma (SSM), and those with a high degree of cumulative sun damage, such as lentigo maligna melanoma (LMM) and desmoplastic melanoma. On the other hand, some melanomas also develop at sun-shielded sites, such as Spitz melanoma, acral melanoma, melanoma arising in congenital or blue naevi, mucosal melanoma (oral, genital, or sinonasal), and uveal melanoma. Nevoid and nodular melanomas may belong to both categories [1]. The histological examination of melanoma allows for the diagnosis and assessing prognostic factors. Pathologists often determine the histological subtypes of melanoma and its growth phase (radial/vertical), as well as histological prognostic features such as the presence of ulceration, Breslow thickness, Clark's level, mitotic rate, lymphovascular invasion, microsatellites, neurotropism, tumor-infiltrating lymphocytes, and tumor regression. Of all these factors, only Breslow thickness and ulceration are included in the most recent American Joint Committee on Cancer classification [5]. This study aims to assess skin melanoma's clinicopathological features and evaluate prognostic factors and their effect on disease outcomes in a Moroccan population.

## Materials and methods

Patient selection

We retrospectively obtained data for 100 patients diagnosed with cutaneous melanoma from 2013 to 2022 in the Department of Pathology of Hassan II University Hospital, Fez, Morocco. Clinical, histopathological, molecular, and follow-up data have been recorded from pathology request forms and the patients' medical records.

Histopathological features

The initial diagnosis of melanoma was made on paraffin-embedded and formalin-fixed blocks after staining with H&E. We assessed the following parameters for all cases: histological subtypes, growth phase (radial/vertical), presence of ulceration, Breslow thickness (measured from the most superficial granular layer of the epidermis or ulcer base to the deepest invasive tumor cell), Clark's level, mitotic count, lymphovascular invasion, neurotropism, tumor-infiltrating lymphocytes, and regression of the tumor.

Immunohistochemistry

On doubtful morphology cases (n=30) where cytoplasmic melanin pigment was not found, immunohistochemical staining was performed using the following antibodies according to the manufacturer's guidelines with an automated stainer: HMB45 (HMB-45) mouse monoclonal antibody, Melan-A (MART-1 /A103) mouse monoclonal antibody, and S-100 (4C4.9) mouse monoclonal antibody.

Mutation analysis

The molecular study was conducted at the molecular pathology unit of our department, examining 43 cases of metastatic melanomas. Our mutation search followed a specific procedure: (1) We highlighted the most representative tumor area on hematoxylin-eosin-Safran (HES)-stained slides where the percentage of tumor cells varied from 40% to over 95%; (2) We conducted a DNA extraction where tumoral DNA was sourced from the paraffin-embedded tumor sections; (3) From the selected formalin-fixed paraffin-embedded (FFPE) tumor block, we retrieved 4-8 sections of 5 μm thickness for DNA extraction using the QIAamp DNA FFPE Tissue Kit (Invitrogen), following the manufacturer's instructions. The DNA concentration (ng/uL) was gauged with a Qubit fluorometer; (4) We analyzed BRAF exon 15 and c-KIT exon 9, 11, 13, and 17 mutations via polymerase chain reaction (PCR) amplification and Sanger sequencing testing; (5) We amplified DNA by PCR (using Master Mix [2X] kits as per the manufacturer's instructions), with forward (5’-ACGAACGAGACTATCCTTTTAC-3') and reverse (5'-CATTGAGTCGTCGTAGAGTCCC-3') primers for the V600E BRAF mutation. The PCR products were then purified using an ExoSAP1 kit in compliance with the manufacturer's instructions. Finally, the purified PCR products were sequenced using direct sequencing with a BigDye Terminator V3.1 Cycle Sequencing Kit (ABI Prism) and analyzed on an Applied Biosystems 3500Dx Genetic Analyzer.

Follow-up

Overall survival (OS) duration was defined as the time between the date of diagnosis and death or the last follow-up visit, both being scored as an event. Disease-free survival was calculated from the date of the diagnosis and the date of the first distant or local disease recurrence, both of which were scored as an event.

Statistical analysis

Statistical analysis was performed using SPSS version 20.0. A descriptive analysis was done, with qualitative variables presented as percentages and quantitative variables as means ± SDs. We used the Chi-squared test to compare two or more percentages. We used the Student's t-test to compare two averages. Survival was described by using the Kaplan-Meier method. Factors associated with outcome were assessed using the log-rank test, with a p-value of less than 0.05 was considered statistically significant.

## Results

A total of 100 cases of cutaneous melanoma were collected during the study period from 2013 to 2022, as summarized in Table 1.

**Table 1:** Clinical and histopathological features of our series. SSM: Superficial spreading melanoma; ALM: Acral lentiginous melanoma; NM: Nodular melanoma; LMM: Lentigo maligna melanoma; TILs: Tumor-infiltrating lymphocytes; BRAF: v-raf murine sarcoma viral oncogene homolog B.

Features	Percentage
Number	100
Age: Mean	65 years
≤60 y	38 (38%)
>60 y	62 (62%)
Sex	
Female	45 (45%)
Male	55 (55%)
Anatomic sites	
Upper limb	6 (6%)
Lower limb	66 (66%)
Trunk	7 (7%)
Head and neck	21 (21%)
Histologic type	
SSM	6 (6%)
ALM	38 (38%)
NM	48 (48%)
LMM	6 (6%)
Desmoplastic	2 (2%)
Breslow thickness	
Mean	10.4 mm
≤1	6 (6%)
1	3 (3%)
2	13 (13%)
> 4	78 (78%)
Ulceration	
Yes	46 (46%)
No	54 (54%)
Clark level	
I	6 (6%)
II	4 (4%)
III	10 (10%)
IV	27 (27%)
V	53 (53%)
Lymphocytes	
Absent	56 (56%)
No Brisk	29 (29%)
Brisk	15 (15%)
BRAF V600E mutation	
Absent	37 (37%)
Present	6 (6%)
Ckit mutation	
Absent	38 (38%)
Present	5 (5%)
Metastasis	
Absent	26 (26%)
Present	74 (74%)

**Figure 1:**
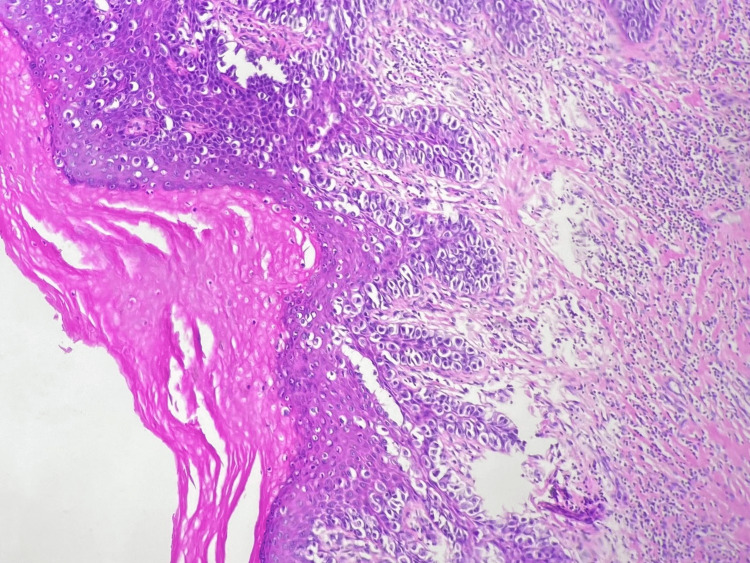
H&E staining of superficial spreading melanoma at x100 magnification.

**Figure 2:**
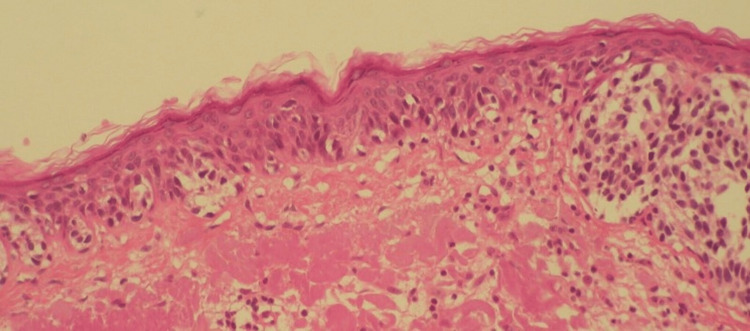
H&E staining of lentigo maligna melanoma (LMM) at x100 magnification.

**Figure 3:**
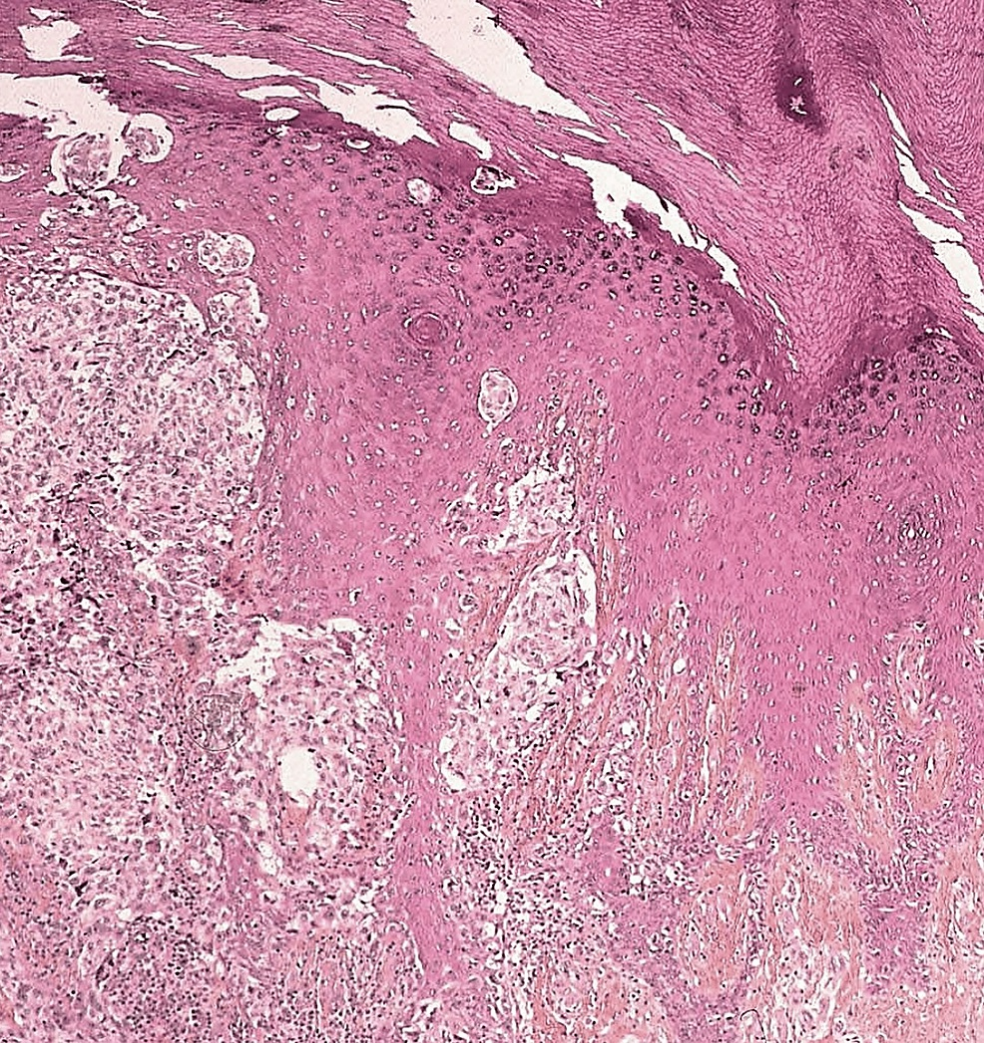
H&E staining of acral lentiginous melanoma at x100 magnification.

**Figure 4:**
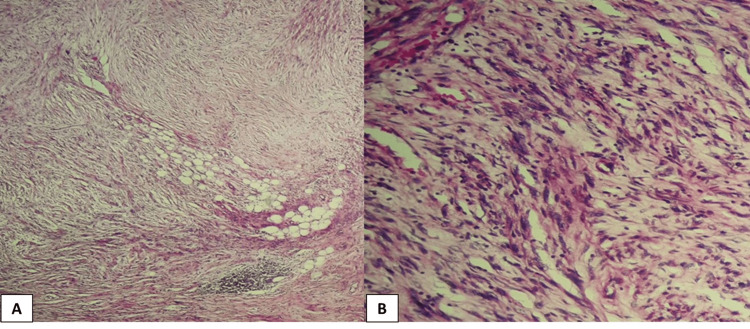
H&E staining of desmoplastic melanoma at x100 (A) and x400 (B) magnifications.

The mean age of our patients was 65 years (range: 22-91 years). The male-to-female sex ratio (M/F) was approximately 1.2. A total of 44% of patients received a consultation for an ulcero-budding tumor with an average estimated delay of 16 months (range: 1-84 months). Lower-limb localization was the most frequent at 66% of cases. Histologically, the nodular (48%) and acro-lentiginous (38%) subtypes were the most common, with Figures 1-4 showing the different histological subtypes found in our series. The average Breslow index was 11 mm (range: <1-53 mm), and 66% of patients had a Breslow index of >4 mm. The presence of ulceration was noted in 46% of cases. The average mitoses was 9/1 mm². A total of 44% of patients had metastatic melanoma at the time of diagnosis, of which 68% (30/44) had only lymph node involvement, and 32% had visceral metastasis, including lung in 24 cases, bone in nine cases, liver in eight cases, and brain in five cases.

**Figure 5:**
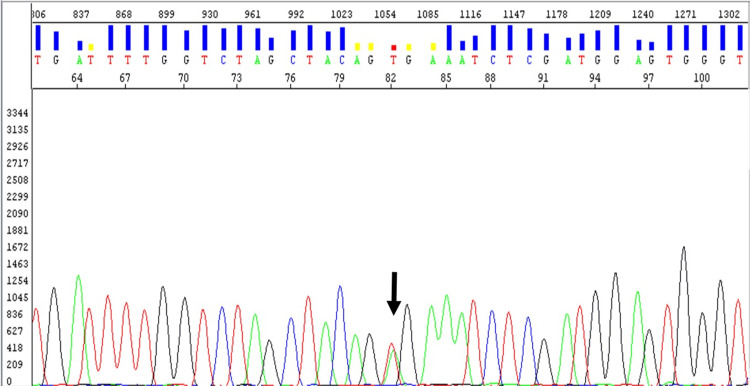
Analysis of exon 15 of the BRAF gene by polymerase chain reaction sequencing (PCR) showing the presence of the c.1760 T>A mutation, p.V600 E.

**Figure 6:**
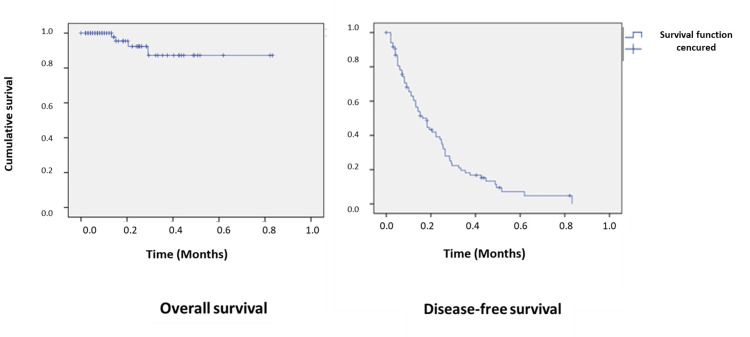
These survival curves show the five-year overall survival and disease-free survival rates in our series.

Immunohistochemical staining was performed in 33 cases using at least two melanic markers (i.e., HMB45, Melan1, and s-100 protein). The analysis of BRAF and C-KIT mutations was performed in 43% of cases of metastatic melanoma and revealed BRAF V600E mutation in six cases (Figure 5) and C-KIT mutation in five cases. Evolution had been marked by the progression of the disease and the involvement of metastases in 74% of cases (i.e., lymph node=74%, pulmonary=46%, cerebral=15%, bone=15%, hepatic=12%, adrenal=6%, and duodenal=6%). The five-year OS and metastasis-free survival rates were 85% and 15%, respectively (Figure 6).

**Figure 7:**
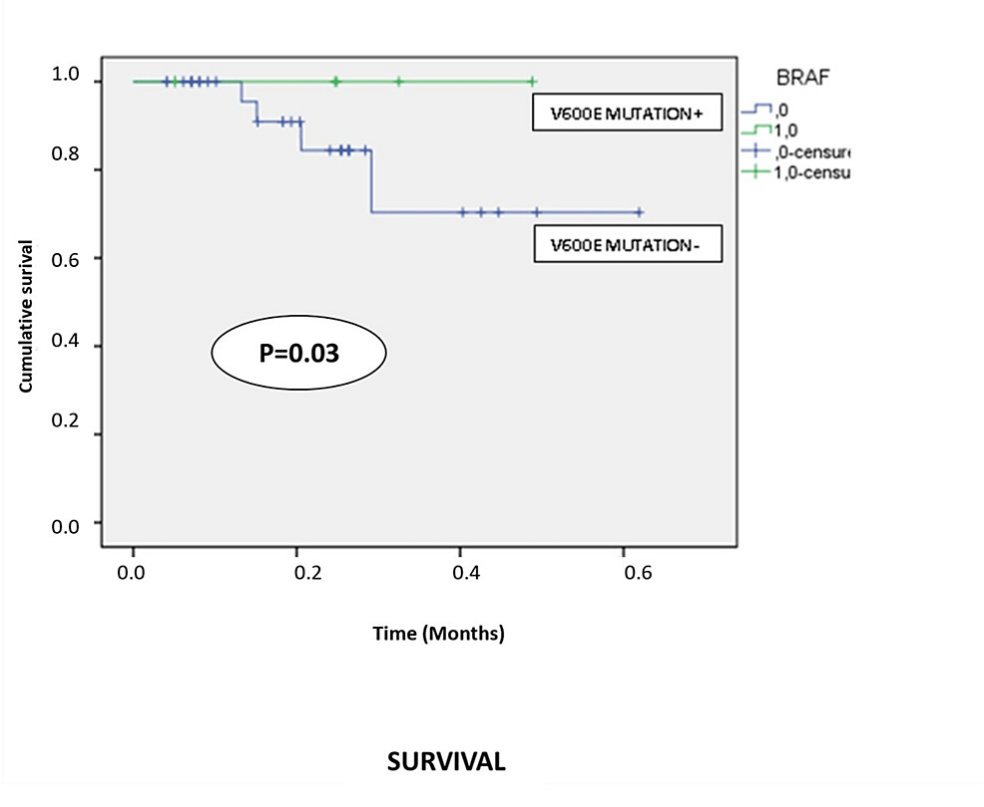
These curves show the difference between patients with the BRAF V600E mutation (green curve) and those without this mutation (blue curve). BRAF: v-raf murine sarcoma viral oncogene homolog B.

There was a significant correlation between Breslow thickness and Clark's level (p<0.001), histologic subtype (p=0.012), and presence of metastasis (p=0.002). Patients with a Breslow index of >4 mm [PW8] [p9] developed metastases in 52.6% of cases, whereas those with a Breslow index of <1 mm did not present metastases in 66.3% of cases (p=0.03). Patients with the BRAF V600E mutation had a better prognosis than those without it (p=0.03) (Figure 7).

We also compared head and neck melanoma features with foot melanoma, as summarized in Table 2.

**Table 2:** Comparison between head and neck, and foot melanoma in our series. SSM: Superficial spreading melanoma; ALM: Acral lentiginous melanoma; NM: Nodular melanoma; LMM: Lentigo maligna melanoma; TILs: Tumor-infiltrating lymphocytes.

		Head and neck melanoma	Foot melanoma	P-value
Number		22	63	
Age				
Mean		65.3 yrs	67.7 yrs	0.045
≤ 60 y		8 (37%)	20 (32%)	
> 60 y		14 (63%)	43 (68%)	
Sex				0.083
Female		13 (59%)	30 (48%)	
Male		9 (41%)	33 (52%)	
Histologic type				<0.0001
SSM		2 (9%)	2 (3%)	
ALM		0 (0%)	37 (59%)	
NM		13 (59%)	24 (38%)	
LMM		7 (32%)	0 (0%)	
Breslow thickness				
Mean		9 mm	16 mm	0.025
≤1		3 (14%)	1 (1.5%)	
1		2 (9%)	1 (1.5%)	
2		4 (18%)	11 (17.5%)	
> 4		13 (59%)	50 (79%)	
Ulceration				<0.0001
Yes		8 (35%)	35 (56%)	
No		14 (65%)	28 (44%)	
Clark level				0.036
I		1 (4.5%)	4 (6.5%)	
II		1 (4.5%)	3 (5%)	
III		3 (14%)	4 (6.5%)	
IV		5 (23%)	23 (36.5%)	
V		12 (54%)	29 (46%)	
Tils				0.022
Absent		13 (59%)	36 (57%)	
No Brisk		6 (27%)	19 (30%)	
Brisk		3 (14%)	8 (13%)	
BRAF V600E mutation				0.04
Absent		6 (27%)	23 (36.5%)	
Present		3 (14%)	0 (0%)	
Ckit mutation				0.033
Absent		6 (27%)	19 (30%)	
Present		1 (4.5%)	4 (6%)	
Metastasis				0.013
Absent		9 (41%)	18 (29%)	
Present		13 (59%)	45 (71%)	

According to these data, a significant difference was noted between melanomas of the head and neck and those of the foot. This was particularly evident in the histological subtype (p<0.0001), and in the presence of ulceration, which was observed in 56% of acral melanomas versus 35% of head and neck melanomas (p<0.0001). The Breslow thickness was greater in acral locations, with 79% of patients showing a thickness of >4 mm (p=0.025). On the other hand, the BRAF V600E mutation was present in nine cases of metastatic melanomas of the head and neck, with three cases testing positive for this mutation. Interestingly, this mutation was not found in any of the 23 cases of acral melanomas tested (p=0.04).

## Discussion

To our knowledge, this is the first and largest Moroccan study specifically describing and analyzing cutaneous melanoma in a single tertiary institution. At present, skin melanoma ranks 17th worldwide in terms of number of cancer cases, accounting for 1.8% of new cases diagnosed in 2020 and 0.6% of death cases [2]. In Morocco, skin melanoma was the 25th most common cancer, with 248 new cases in 2020, contributing 0.42% of new cases and 114 (0.32%) death cases [3]. Similar to data reported in the literature, the mean age of our patients was 65 years (range: 22-91 years), with a male predominance and an M/F ratio of approximately 1.2 [6,7]. However, Alshedoukhy A et al. [8] reported a female predominance and a younger average age in their study. According to data from the literature [9,10], the lower limbs are the most common localization of skin melanoma, which is similar to our findings. In our study, the most frequent localization site of skin melanoma observed in the lower limb was the sole, followed by the head and neck. Sparks DS et al., who reported 107 patients with primary scalp melanoma, also supported the hypothesis that primary melanoma of the scalp represents a unique aggressive subtype with high disease rates and poor disease and survival outcomes [11]. According to the histopathological classification of melanomas, there are four main subtypes: ALM, NM, LMM, and SSM. These four subtypes are reported at variable frequencies depending on the series. In our study, as well as in those of Alshedoukhy A et al. [8] and Sharma N et al. [12], the most common histological type diagnosed was NM, followed by ALM. In contrast, Luk NM et al. [13] and Nam KW et al. [14] reported ALM as the most common subtype in their studies, followed by NM, whereas Baykal C et al. [15] found that the most common subtype was SSM, followed by LMM (Table 3). In our study, the maximum Breslow thickness (up to 4 mm) was found in 78% of cases, 42% of which were NMs. The frequency of thick melanomas (Breslow index >4 mm) in our study reflects not only a delay in diagnosis but also the general public's lack of awareness regarding pigmented lesions. As a result, Breslow's thickness was considered the most critical histo-prognostic factor for the occurrence of metastatic melanoma. Such a result confirms the conclusions of various authors since the description of this variable by Breslow in 1970 [16]. It is thought that the thicker lesions may represent more advanced tumors, with more intrinsic biological aggressiveness, than those with radial growth only [17]. Similar findings have been reported by others cited in Table 3.

**Table 3:** Clinical and histopathological features of reported melanoma series. SSM: Superficial spreading melanoma; ALM: Acrolentiginous melanoma; NM: Nodular melanoma; LMM: Lentigo maligna melanoma.

		Our cohort 2023	Sharma N et al. (2021) [12]	Alshedoukhy A et al. (2019) [8]	Baykal C et al. (2016) [15]	Nam KW et al. (2015) [14]	Luk NM et al. (2004) [13]
Number		100	44	98	227	100	63
Age (years)	Mean (years)	65	61.29	58	57.7	55	61.5
	≤60	38%	43%	41%	_	39%	43%
	>60	62%	57%	59%	_	61%	57%
Sex							
	Female	45%	47.73%	57%	51%	64%	43%
	Male	55%	52.27%	43%	49%	36%	57%
Anatomic site							
	Upper limb	6%	12%	5%	12%	18%	8%
	Lower limb	66%	53%	45%	29%	60%	71.5%
	Trunk	7%	10%	2%	24%	11%	9.5%
	Head and neck	21%	20%	20%	35%	11%	11%
Histologic type							
	SSM	6%	11%	7%	38%	9%	19%
	ALM	38%	39%	27%	20%	58%	50.8%
	NM	48%	41%	38%	7%	29%	27%
	LMM	6%	9%	5%	32%	4%	3.2%
	OTHERS	2%	0%	0%	3%	0%	0%
Breslow Thickness (mm)							
	Mean	10.4 mm					
	≤1	6%	26%	1%	31%	27%	17.5%
	1	3%	15%	6%	24%	13%	14.3%
	2	13%	7%	7%	25%	28%	23.8%
	>4	78%	52%	47%	20%	32%	44.4%
Ulceration							
	Yes	46%	48%	65%	_	_	89%
	No	54%	52%	35%	_	_	11%
Clark level					_		
	I	6%	7%	7%	_	14%	0%
	II	4%	11%	7%	_	10%	8%
	III	10%	30%	5.5%	_	15%	12.7%
	IV	27%	22%	40%	_	38%	51%
	V	53%	30%	36%	_	23%	28.3%
Lymphocytes					_		
	Absent	56%	41%		_	_	_
	Not brisk	29%	45%		_	_	_
	Brisk	15%	14%		_	_	_
Metastasis			_		_	_	_
	Absent	26%	47%	47%	_	_	_
	Present	74%	53%	53%	_	_	_

Determining BRAF mutation status is essential for the therapeutic management of melanomas, particularly those of the skin. Anti-BRAF therapies are associated with significantly improved OS in patients with metastatic melanoma and the BRAF V600E mutation. Our study found the BRAF V600E mutation in six cases among 43 metastatic melanomas. These patients had a better prognosis than those without the mutation. In a recent meta-analysis, BRAF mutations were associated with histological subtype and tumor site but not with patient age or sex. They were frequently detected in patients with SSM and MMs arising in non-chronically sun-damaged skin [18]. In a large, population-based national study using US cancer registries from the Surveillance, Epidemiology, and End Results 13 Registries (SEER-13) database, Lachiewicz AM et al. compared the prognosis of patients with head and neck melanomas with that of patients with melanomas at other sites [19]. Similar to our results (Table 2), they reported that there was a notable survival difference between head and neck melanoma and melanoma of other sites, even after adjusting for important prognostic factors.

## Conclusions

The incidence of cutaneous melanomas has been increasing in recent years. Unfortunately, in our context, the diagnosis is made at an advanced stage, and this is because of the delay in diagnosis and the neglect of the monitoring of pigmented lesions. On the other hand, the predominant acral localization also explains this delay in the diagnosis of incipient lesions inaccessible to self-detection by the patient. However, prospective and nationwide studies are needed to better understand the characteristics of cutaneous melanoma in our population. Awareness campaigns and early detection of melanoma remain the main tools that can guarantee the early diagnosis and curative treatment of melanoma and prevent the occurrence of metastasis.
